# Interaction Effects Do Not Consistently Improve the Transferability of Species Distribution Models

**DOI:** 10.1002/ece3.74362

**Published:** 2026-09-13

**Authors:** Duarte S. Viana, Laura Cardador, Miguel Clavero

**Affiliations:** ^1^ Estación Biológica de Doñana, CSIC Seville Spain; ^2^ Departament de Biologia Evolutiva, Ecologia i Ciències Ambientals, Facultat de Biologia Universitat de Barcelona (UB) Barcelona Spain; ^3^ Institut de Recerca de la Biodiversitat (IRBio) Universitat de Barcelona (UB) Barcelona Spain

**Keywords:** machine learning, niche, non‐stationarity, predictive ability, SDM, statistical interactions

## Abstract

Species responses to the environment are often non‐stationary, varying in space and time depending on the environmental context. This is one of the reasons why species distribution models (SDMs) often have low transferability. Context dependence may arise due to frequent and widespread interactive effects of environmental drivers on species occurrence. Thus, accounting for interactions in SDMs could in principle improve their transferability. Alternatively, incorporating driver interactions may lead to overfitting issues and reduce model transferability. We systematically assessed the extent to which interactions improve model transferability across taxa and biomes. We fitted MaxEnt and Boosted Regression Trees (BRT) models to presence data of 1158 species and evaluated their transferability to (1) independent native areas of plants, reptiles, birds and bats, (2) exotic ranges of plant species and (3) exotic ranges of bird species. While the performance of both model types (MaxEnt and BRT) was highly correlated, gains or losses in transferability when incorporating interactions were not consistent between model types. Incorporating interactions did not have any consistent impacts on transferability in 84% of the models according to a concordance (between model types) and significance criterion. In the remaining models, similar percentages of models had improved and worse transferability (~8% each). We did not detect any relevant effects of taxon, dataset, sample size, sampled area and environmental novelty on transferability gains when incorporating predictor interactions. Overall, uninformed predictor interactions modelled via widely used machine learning algorithms did not increase SDM transferability, suggesting that modelling routines should carefully consider their specification and usefulness for model extrapolation.

## Graphical abstract

Species distribution models (SDMs) are a widely used tool in ecology, macroecology, biogeography and conservation biology. We provide the first systematic evaluation of how the incorporation of predictor interactions in SDMs affects their transferability across a wide range of taxa and regions.

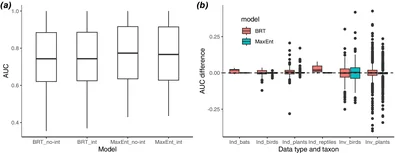

## 1 Introduction

Understanding and predicting how species respond to environmental variation and change is of primary importance for ecology, biogeography and conservation biology. This is why there is a huge body of work aimed at developing and improving species distribution models (SDMs; also known as ecological niche models). However, the predictive ability and transferability of such models is typically limited (Liu et al. 2020), because, in addition to sampling and analytical issues, the species' responses to the environment are non‐stationary and context dependent in space and time.

Evidence for changes in the realised niche of species in space and time has been accumulating in different ecosystems (Avidad et al. 2025; Di Marco et al. 2021; Pearman et al. 2008; Pulliam 2000; Zachariah Atwater and Barney 2021; Zurell et al. 2024). Invasive species, in particular, are especially prone to non‐stationarity, as they encounter a diversity of contexts and environments in recipient ecosystems (Cardador and Blackburn 2020; Guisan et al. 2014; Viana et al. 2023; Zachariah Atwater and Barney 2021). Non‐stationarity, assessed as variation in the response of a species to a given environmental gradient, often arises when species responses to the environment are context dependent (Catford et al. 2022). The abiotic context (e.g., climate) in a region at a given time can influence the response of species to any particular environmental gradient. For example, temperature‐dependent body size has been shown to alter species' responses to global warming (Lindmark et al. 2018); and regional microclimate has been shown to moderate plant species' responses to global warming (De Frenne et al. 2013). The effect of climate can also override the effect of a regional environment that would otherwise be stronger, or interact with a regional environment (e.g., land cover) by changing how species exploit it. An extreme example comes from Japan, where the invasive giant bamboo ( *Phyllostachys bambusoides* ) reverses its response to the canopy cover depending on temperature—positive under cool conditions and negative under warm conditions—resulting in an interaction between light and temperature (Spake et al. 2021). Indeed, much of the observed context dependence can result from interactive effects between environmental drivers (e.g., climate and land cover in the giant bamboo case). As such, we could expect that modelling driver interactions generally improves the predictability of species distributions.

Species Distribution Models are widely used to model species responses to the environment and predict their distributions under past, present and future environmental conditions (Elith and Leathwick 2009). These models can incorporate interactive effects between environmental predictors, but informed decisions to include them are rarely made. Depending on the study goals and knowledge of the species and ecosystems, approaches based on the general linear model (e.g., GLM, GAM) often omit interactions between multiple predictors due to the overwhelming complexity of specifying them as well as potential overfitting issues caused by the expansion of estimated effects. Machine learning models (e.g., MaxEnt, RF, BRT), on the other hand, are typically used to model flexible non‐linear and interactive effects learned from the data, but can also be prone to overfitting if the algorithms are not carefully tuned (Elith et al. 2006).

The question is whether accounting for interactive effects improves the transferability of SDMs, especially when predicting (extrapolating) for different regions and times. On one hand, predictor interactions can account for spatiotemporal variation in niche responses, and thus improve the predictability of species distributions. Although direct tests remain elusive, models and algorithms that allow fitting flexible interactive effects, such as machine learning algorithms, have been systematically proven to achieve higher transferability (Elith et al. 2006; Heikkinen et al. 2012; Tsoar et al. 2007; Valavi et al. 2022, 2023). On the other hand, incorporating interactive effects can produce overfitting of training data and reduce the predictability of species distributions if the interactions themselves are regionally idiosyncratic or not pervasive across the species' ranges—i.e., non‐stationarity produced by complex environmental interactions, fluctuations and change. Moreover, methodological issues might hamper the estimation of relevant interactive effects, including sampling and modelling limitations, omission of important predictors, and scale mismatches (Rousseau and Betts 2022; Takolander et al. 2025; Yates et al. 2018). Therefore, allowing and specifying interactive effects in SDMs is not a straightforward decision.

To date there is no systematic exploration of the extent to which interactive effects improve the transferability of SDMs across taxa and spatiotemporal contexts. Here, we make a first exploration using data specifically collected to test the predictive ability of SDMs for plants, reptiles, birds and bats (presence‐only species records for model training, and independent presence‐absence records for model evaluation) as well as native and exotic distributions of a wide range of plant and bird species. Because SDMs typically model the effects of numerous predictors, which complicates the specification of relevant interactions, we chose two commonly used machine learning algorithms—MaxEnt and BRT—with which the interactive effects can be easily switched on or off. These methods also allowed us to avoid having to specify multiple interactions for each of the hundreds of species included in the study. We expected that allowing flexible interactive effects in SDMs would in general increase model transferability by more efficiently modelling environmental context. Alternatively, similar or decreased model transferability would indicate either overfitting issues or other biological and methodological issues as mentioned above. To pin down potential factors explaining differences in model transferability driven by predictor interactions, we further related these transferability differences to the type of model (Maxent vs. BRT), extrapolation setting (depending on the dataset), taxonomic group, the number of presence points in the training data, the area encompassed by the training occurrences and the environmental novelty between training and testing areas (i.e., the level of extrapolation; low within native areas and generally higher for exotic ranges). We point out that the methodology of our study was not appropriate for linking the estimated interactive effects to the underlying ecological processes causing context dependence. Rather, the goal was to test whether machine‐learned statistical interactions between predictors could generally improve model transferability—by somehow predicting the effect of context‐dependent environmental conditions determining the geographical distributions of species.

## 2 Methods

### 2.1 Data

**FIGURE 1:**
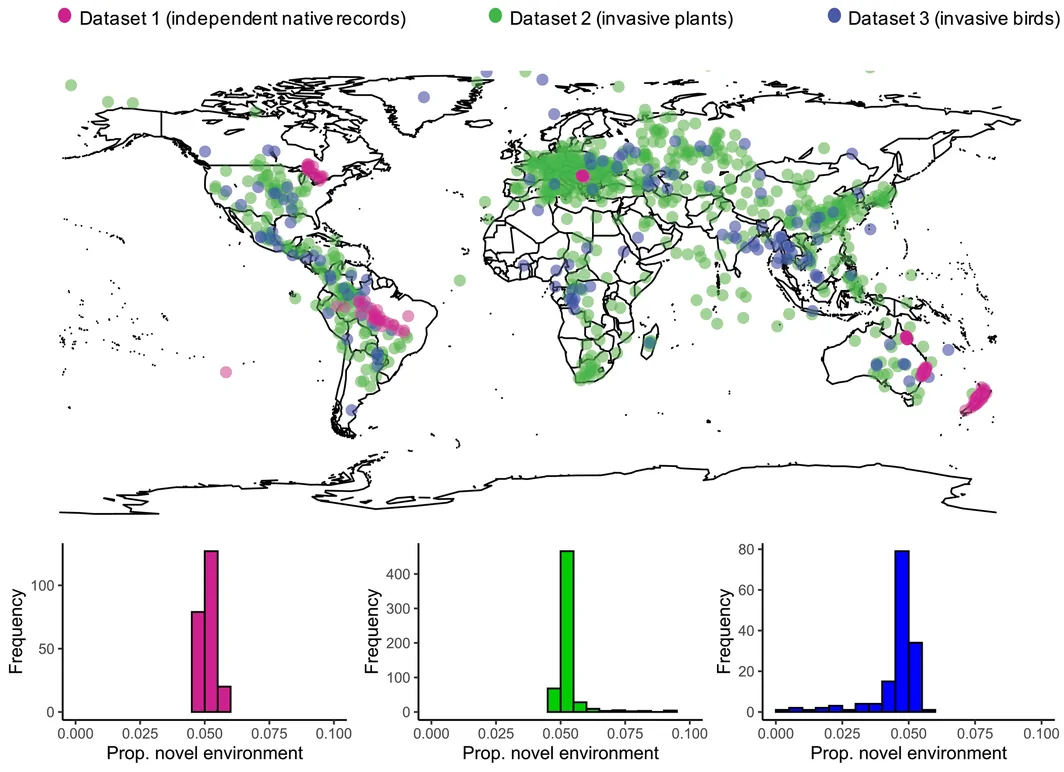
Geographical distribution of the training data and the frequency distribution of the environmental novelty (i.e., proportion of novel environment) in the testing area (bottom histograms). We show the centroid of the minimum convex polygon encompassing the training presence data for each species in each dataset, corresponding to a total of 1158 species.

With the idea of making a general, systematic exploration of the impact of predictor interactions on model transferability, while keeping computational feasibility and result tractability, we selected three case studies covering a wide range of taxa and regions (Figure 1). Data filtering and preparation for modelling were mostly kept as in the original studies, such that the main modelling decisions we made were the type and parametrisation of SDMs.

The first dataset is the result of a collaborative effort to collect data with the purpose of comparing the performance of different species distribution modelling approaches (Elith et al. 2020). This dataset comprises data of 226 anonymised species, including plants, reptiles, birds and bats, collected across six regions of five continents. For each species, the data records are divided in a training dataset with presence‐only records and respective background locations, and a testing dataset with presence‐absence records from an independent survey for evaluating model performance. All species records were matched to predictor variables deemed relevant for each taxon, including climatic, topographical, physico‐chemical, among other predictors (see the detailed data description in Elith et al. 2020). These data were accessed via the R package *disdat* (Elith et al. 2020).

The second dataset corresponds to a data compilation of ca. 14 million presence records of 1135 plant species in native and exotic ranges across the entire world (Zachariah Atwater and Barney 2021). The species occurrences were collated mainly from GBIF, but data from the United States were complemented with records from the Early Detection and Distribution Mapping System (Early Detection and Distribution Mapping System, http://www.eddmaps.org). All records were matched to four climatic predictors resulting from a PCA on bioclimatic variables from the WorldClim Global Climate Database (Hijmans et al. 2005). The background locations (10,000 for each species) were randomly chosen from the pool of presence records of all species within the respective biogeographic regions. All the data, including the species occurrences and predictors, were obtained from the original study. The SDMs were fitted to native occurrence records and used to predict the exotic distribution. We retained a total of 787 plant species for analysis according to a model quality criterion (see below).

The third dataset is a compilation of presence records of 150 invasive bird species in their native and exotic ranges at 0.083° resolution from the GBIF database (Cardador and Blackburn 2020). The background locations in native ranges consisted of 10,000 pseudo‐absences randomly drawn from all biomes occupied by each species across its native ranges. In the exotic range, the background area was defined as the total area each species could have potentially colonised, based on the year of introduction and an estimated invasion speed of 4.59 km/year (Blackburn et al. 2009). For this dataset, in addition to climatic data extracted from the WorldClim database (Hijmans et al. 2005), we used land use predictors which were not included in the original study to model potential habitat‐climate interactions. Land use data, extracted from the Land‐Use Harmonization2 dataset (Hurtt et al. 2020), correspond to fractional land‐use patterns, the transitions between different land uses, and important agricultural management details at a spatial resolution of 0.25 × 0.25°. Both climate and land use variables were averaged across the period 1970–2000. The final set of predictor variables, selected to avoid collinearity issues, was: maximum temperature of the warmest month, minimum temperature of the coldest month, precipitation of the driest month, precipitation of the wettest month and precipitation seasonality as climatic variables, and forest, non‐forest, pasture, crop and urban cover as land use variables (land use subcategories were aggregated). We excluded two species due to model fitting issues.

### 2.2 Species Distribution Models

We chose MaxEnt and Boosted Regression Trees (BRT), two widely used machine learning approaches, that allow an optional estimation of interactive effects without having to specify them. Furthermore, machine learning algorithms are ideal for modelling nonlinear relationships, also preventing spurious interactions that could arise from nonlinearity (Duncan and Kefford 2021). Two versions of each model type were applied to all species: models either with or without predictor interactions. MaxEnt models were run using the R package *dismo* (Hijmans et al. 2017) always allowing linear and quadratic model terms (called “features” in MaxEnt), and activating or deactivating the product (i.e., interaction) feature. BRT were run using the R package *gbm* (Greenwell et al. 2019) with a learning rate (i.e., “shrinkage”) of 0.01, 1 k trees, a cross validation procedure with five folds and bag fraction of 0.75, and an interaction depth of either 1 (no interactions) or 2 (two‐way interactions), using the same cross‐validation data folds in both model fits. Even though the two approaches deal with complexity and fit the data in different ways, including the estimation of interaction effects, they are commonly used and combined within the same SDM frameworks and studies (Araújo and New 2007). Our goal was to exclusively compare differences in transferability between models with or without predictor interactions (while keeping the remaining parameters fixed), independently of likely differences in their ecological interpretations. Models showing poor fits to the training data—i.e., those showing in‐sample goodness‐of‐fit of < 0.70 (Hosmer et al. 2013; Pearce and Ferrier 2000), as measured by the area under the receiver operating curve (AUC) – were excluded, resulting in 1158 species kept for analysis. For each species, we assessed the predictive ability (or transferability) of the models by calculating the out‐of‐sample AUC with the testing data, consisting of either the independent evaluation data in the first dataset or the occurrence data in the exotic range in the other two datasets. We then compared the out‐of‐sample AUC of models with and without interactions across species.

### 2.3 Model Comparison

To evaluate the effect of accounting for predictor interactions on model transferability, we first calculated the difference in out‐of‐sample AUC between models with and without interactions, such that more positive values indicate a better predictive ability of models with interactions. The significance of this difference was evaluated using the Delong test for concordance in Receiver Operating Curves (ROC) using the function “roc.test” of the R package *pROC* (Robin et al. 2011). To generally test for differences in the transferability of models with and without interactions, we performed paired permutation tests for each species and model separately using the function “twoSamplePermutationTestLocation” of the R package *EnvStats* (Millard 2013). Then, using a linear model, we modelled the AUC difference as a function of model type (Maxent vs. BRT) in interaction with the case study (3‐level factor), the number of presence points in the training data, the area encompassed by the training occurrences, and the environmental novelty between training and testing areas (calculated for each species distribution as the proportion of Mahalanobis distances above the 0.95 quantile of their frequency distribution). Because the number of features that MaxEnt uses (including the “product” feature) depends on the sample size, the number of presence records also corrects for possible biases produced by species with lower data availability (25% of the species had less than 100 training occurrences). In addition, we repeated this analysis for each case study and model type separately to account for differences in test settings and algorithms (Supporting Information, Tables S1–S9). For the first case study, we also tested for differences between taxonomic groups in interaction‐induced transferability differences.

## 3 Results

**FIGURE 2:**
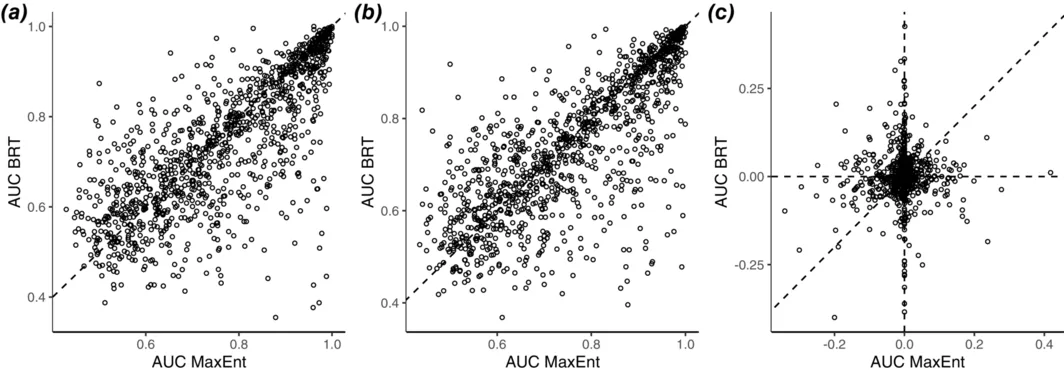
Relationship between the transferability (AUC) of MaxEnt and BRT. (a) AUC of models without interactions. (b) AUC of models with interactions. (c) AUC difference (AUC of models with interactions minus AUC of models without interactions). Dashed lines in panels (a) and (b) represent the 1:1 line (equal transferability between model types), and in panel (c) represent the 1:1 line (if AUC difference would be perfectly consistent between model types) and the no difference “0” lines (when the incorporation of interactions does not produce any difference in model transferability).

The general predictive performance of the models was moderate (according to Hosmer et al. 2013; Pearce and Ferrier 2000), with a global out‐of‐sample AUC median of 0.756 (Q5% = 0.501, Q95% = 0.986; range: 0.355–1). MaxEnt models had slightly better performance compared to BRT, with median AUC of 0.771 and 0.743, respectively. As expected, their performances were strongly correlated, both for models with and without predictor interactions (*r* = 0.77 and 0.78, respectively; Figure 2a,b). However, the difference in performance between models with and without interactions was not systematically consistent between MaxEnt and BRT (*r* = 0.05; Figure 2c).

**FIGURE 3:**
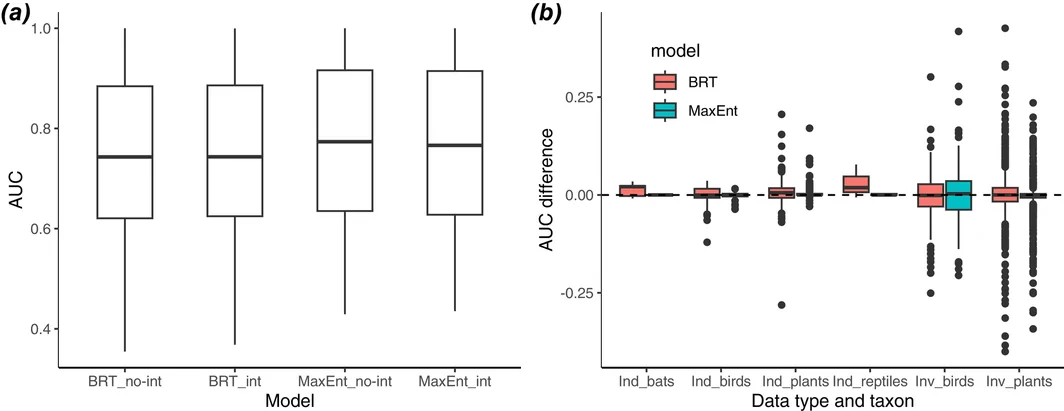
Comparison of transferability across model types (MaxEnt vs. BRT) and models with versus without interactions (a), as well as across taxa and data types (b). AUC difference is given by the AUC of models with interactions minus the AUC of models without interactions. no‐int: Without interactions; int.: With interactions; Ind: Independent records in native region (dataset 1); Inv: Invasive species (datasets 2 and 3).

In general, we did not detect an overall difference in the predictive performance of SDMs when including predictor interactions. The difference between the AUC of models with and without interactions was not significant (*p* = 0.42 according to a paired permutation test) (Figure 3a), even when accounting for the test setting (i.e., predicting with independent records within the native or invaded region) and taxonomic group (Figure 3b). Gains or losses in transferability were more pronounced when predicting in exotic ranges compared with native ranges, despite the general lack of difference between models with and without interactions (Figure 3b). Pairwise Delong's classification for concordance in AUC (mean ± SD of AUC difference = −0.003 ± 0.051 for MaxEnt and 0.001 ± 0.062 for BRT) support a slight tendency for improvement (over worsening) in transferability (21% and 29% improvement, and 24% and 24% worsening for MaxEnt and BRT, respectively). However, when considering only consistent differences between MaxEnt and BRT, similar percentages of improvement (8.1% of the species) and worsening (8.5% of the species) were observed. This low percentage of consistent significant differences between models with and without interactions indicates that in general the inclusion of interactive effects does not significantly influence model performance. The difference in AUC was not explained by either of the considered factors (Table 1, Tables S1–S9), not even when considering the subset of models with consistent improvement or worsening. The few observed significant effects were inconsistent across the different model variations (Table 1, Tables S1–S9), and were mostly driven by a few outlier values. Overall, we did not detect any consistent effects, as also supported by the models' lack of explanatory power (*R* ^2^ < 0.11 in all models).

**TABLE 1:** Results of the model of AUC difference (*R* ^2^ = 0.013).

Model term	Estimate ± SE	*t*	*p*
Intercept	−0.021 ± 0.014	−1.482	0.138
Study_exotic_birds	−0.008 ± 0.007	−1.214	0.225
Study_exotic_plants	−0.009 ± 0.005	−1.652	0.099
Model_MaxEnt	−0.002 ± 0.005	−0.363	0.716
Number_presences	−0.000 ± 0.001	0.390	0.696
Prop_novel_environment	0.424 ± 0.226	1.878	0.061
Area (log‐transformed)	0.000 ± 0.001	0.499	0.618
Study_exotic_birds: Model_MaxEnt	0.005 ± 0.008	0.601	0.548
Study_exotic_plants: Model_MaxEnt	0.003 ± 0.006	−0.424	0.672

*Note:* The first case study (test data in native area) and BRT models are the reference levels (intercept) for the case study and model type effects, respectively. The model included the ΔAUC derived from all performed SDMs (*N* = 2318). The colon indicates an interaction effect. Model residuals were normal.

## 4 Discussion

In general, modelling interactive effects did not influence the transferability of the fitted species distribution models, not even within the native distributions of species where environmental conditions are expected to be more similar than in distant invaded regions. In fact, the models' predictive power was often quite low, revealing a high degree of statistical non‐stationarity potentially explained by biological factors such as high environmental heterogeneity, global environmental change and the plasticity of species responses to environmental variation, as well as analytical issues such as omission of important predictors and sampling scale and coverage (Yates et al. 2018).

Environmental heterogeneity, exacerbated by rapid global change, poses significant challenges for an appropriate characterisation of the diversity of environmental contexts under which species live, hindering our ability to capture biologically relevant environmental interactions. For example, global change is disrupting the species' responses to the environment and causing extinction debts, producing environmental decoupling that further complicates model training and transferability (Viana and Chase 2022; Zurell et al. 2024). Moreover, differences in the regional biotic context and landscape connectivity can affect the species' responses to the abiotic environment. For example, the presence of new competitors or predators can affect the realised niche response (Alexander et al. 2015; Brooker 2006; Viana et al. 2023), and even reverse responses (Suttle et al. 2007); and differences in connectivity among regions can lead to differences in the ability of populations to track their niche, causing variable niche responses across the species' ranges (Schloss et al. 2012).

How species respond to the environment is also mediated by the species' functional traits—which can evolve and be more or less plastic, generating the high intraspecific trait variability observed in many species. This biological variation and plasticity introduce an additional layer of variation in species–environment interactions that further compromises model transferability (Paton and Matthiopoulos 2016). For example, body size is a critical ecophysiological factor that can mediate differential population responses to climatic gradients, and behavioural variability in animals can lead to differential selection of microhabitats and diets. This is why there have been efforts to model distributions at the subspecies level (Cardador et al. 2022; Oney et al. 2013) or to develop process‐based SDMs that integrate intraspecific trait variability (Chardon et al. 2020).

In addition to ecological and biological factors, analytical issues, such as spatial biases in data collection, low sample size and omission of important environmental drivers (Sequeira et al. 2016), may also explain why models incorporating interactive effects were not more transferable. Usually, species are not sampled across the entire environmental gradients on which they occur (or could occur given their fundamental niche), which precludes an efficient characterisation of the variety of environmental contexts where populations subsist. Furthermore, data quality is often affected by differences in the resolution of environmental variables that can potentially miss important variation, as well as by a misalignment between the species' response and the measured environmental grain (Lu and Jetz 2023). These scaling issues can get even worse when modelling interactive effects, since their effective estimation should depend on data resolutions fine enough to capture biologically relevant interdependencies.

Lastly, the methodological approach also affects how interactive effects are modelled. Our results demonstrate that the improvement or worsening in model transferability when including interactive effects is not consistent between models (in this case, MaxEnt vs. BRT). This is possibly caused by the specific algorithms behind each model, but it is difficult to dissect differences in model results because the incorporation of interactive effects produced few significant differences and inconsistent trends. When interactions are deemed important, a careful evaluation of model results should guide model choice and specification. A model averaging approach could also be a possibility to smoothen differences in model fitting, including the effect of interactions, although the observed inconsistency between models calls for careful consideration of how predictor interactions are modelled.

This study was a first systematic exploration of whether accounting for machine‐learned predictor interactions in SDMs could improve model transferability by better predicting context‐dependent responses of species to the environment. The generalised poor ability of predictor interactions to improve the predictability of species distributions hindered our goal of identifying general circumstances under which predictor interactions should be specified in SDMs. This is likely the result of non‐stationary species responses combined with methodological challenges to specify ecologically relevant models. Until further systematic examinations are conducted, interaction effects should be carefully specified and evaluated in modelling pipelines according to cross‐validation procedures and inspection of model transferability.

## Author Contributions

**Duarte S. Viana:** conceptualization (lead), data curation (supporting), formal analysis (lead), funding acquisition (lead), investigation (lead), methodology (lead), project administration (lead), resources (equal), validation (lead), visualization (lead), writing – original draft (lead), writing – review and editing (equal). **Laura Cardador:** conceptualization (supporting), data curation (lead), writing – review and editing (equal). **Miguel Clavero:** conceptualization (supporting), funding acquisition (supporting), project administration (supporting), resources (supporting), supervision (lead), writing – review and editing (equal).

## Funding

This work was supported by Agencia Estatal de Investigación, European Commission, MSCA Project 101059418, and project PID2023‐150807NA‐I00 funded by MICIU/AEI/10.13039/501100011033 and European Regional Development Fund, UE.

## Conflicts of Interest

The authors declare no conflicts of interest.

## Supporting information


**Data S1:** ece374362‐sup‐0001‐Supinfo1.pdf.

**Table S1:** Results of the model of AUC difference for the first case study (testing with independent data from native range) (*R* ^2^ = 0.053).
**Table S2:** Results of the model of AUC difference for the second case study (testing with data from plants' exotic range) (*R* ^2^ = 0.003).
**Table S3:** Results of the model of AUC difference for the third case study (testing with data from birds' exotic range) (*R* ^2^ = 0.000).
**Table S4:** Results of the model of AUC difference resulting from BRT predictions for the first case study (testing with independent data from native range) (*R* ^2^ = 0.107).
**Table S5:** Results of the model of AUC difference resulting from BRT predictions for the second case study (testing with data from plants' exotic range) (*R* ^2^ = 0.015).
**Table S6:** Results of the model of AUC difference resulting from BRT predictions for the third case study (testing with data from birds' exotic range) (*R* ^2^ = 0.050).
**Table S7:** Results of the model of AUC difference resulting from MaxEnt predictions for the first case study (testing with independent data from native range) (*R* ^2^ = 0.063). The bats taxon is the reference level (intercept) for the taxon effects.
**Table S8:** Results of the model of AUC difference resulting from MaxEnt predictions for the second case study (testing with data from plants' exotic range) (*R* ^2^ = 0.012).
**Table S9:** Results of the model of AUC difference resulting from MaxEnt predictions for the third case study (testing with data from birds' exotic range) (*R* ^2^ = 0.000).

## Data Availability

All data used can be openly accessed from the cited publications and repositories, and the code required to reproduce the analyses is available as Supporting Information.
