# Supplementary material for: Interaction Effects Do Not Consistently Improve the Transferability of Species Distribution Models
Source: Ecol Evol. 2026 Sep 13;16(9):e74362. doi: 10.1002/ece3.74362 (PMC13572786; doi:10.1002/ece3.74362)
Supplement: Supplementary file 1 [file ECE3-16-e74362-s001.pdf]

```
##########################################################
# Data analysis of the manuscript:                          #
# "Interaction effects do not consistently improve the transferability   #
# of species distribution models"                           #
#########################################################

# Load libraries
library(sf)
library(sfheaders)
library(terra)
library(geodata)
library(elevatr)
library(rSDM)
library(maps)
library(mapdata)
library(dplyr)
library(tidyr)
options(java.parameters = "-Xmx8g")
library(dismo)
library(gbm)
library(ROCR)
library(pROC)



#####################################################################
#####################################################################

# Data from Cardador et al. 2019 (https://doi.org/10.1111/geb.13166)

###################################

# DON'T RUN AGAIN
# Prepare data for all species

# Land use
lu <- terra::rast("states.nc")
lu <- lu[[time(lu)>=1970 & time(lu)<=2000]]
lu.var <- varnames(lu)

# Climate data
clim <- worldclim_global(var='bio', res=10,
           path="/Users/viana/Dropbox/Paper_niche_spatial-change/WorldClim")

# Extract data for all species
sp.list <- list.files()
sp.data <- list()
```

```
for(s in 1:length(sp.list)){
  sp <- read.table(sp.list[s], header=T, sep="\t")
  occ.sf <- st_as_sf(x = sp, coords = c("x", "y"), crs = "+proj=longlat +datum=WGS84")
  # Land use
  luv <- lu["primf"]
  luv <- mean(luv, na.rm=TRUE)
  lu.sp <- terra::extract(luv, occ.sf)
  if(length(which(is.na(lu.sp[,2])))>0){
    moved <- points2nearestcell(occ.sf[which(is.na(lu.sp[,2])),], luv, table=FALSE,
map="none")
    lu.sp2 <- terra::extract(luv, moved)
    lu.sp[which(is.na(lu.sp[,2])),2] <- lu.sp2[,2]
  }
  sp <- cbind(sp, lu.sp[,-1])
  names(sp)[ncol(sp)] <- "primf"
  for(v in lu.var[-1]){
    luv <- lu[v]
    luv <- mean(luv, na.rm=TRUE)
    lu.sp <- terra::extract(luv, occ.sf)
    if(length(which(is.na(lu.sp[,2])))>0){
      lu.sp2 <- terra::extract(luv, moved)
      lu.sp[which(is.na(lu.sp[,2])),2] <- lu.sp2[,2]
    }
    sp <- cbind(sp, lu.sp[,-1])
    names(sp)[ncol(sp)] <- v
  }
  # Climate
  clim.sp <- terra::extract(clim, occ.sf)
  names(clim.sp) <- substr(names(clim.sp),11,nchar(names(clim.sp)))
  if(length(which(is.na(clim.sp[,2])))>0){
    moved <- points2nearestcell(occ.sf[which(is.na(clim.sp[,2])),], clim, table=FALSE,
map="none")
    clim.sp2 <- terra::extract(clim, moved)
    clim.sp[which(is.na(clim.sp[,2])),-1] <- clim.sp2[,-1]
  }
  sp <- cbind(sp, clim.sp[,-1])

  # Data
  sp.data[[s]] <- sp
}

###################################
```

```
# SDMs

predictabil1 <- data.frame(region=rep("Cardador",150), sp=lau$Sp, me01=NA, me02=NA,
                          me1=NA, me2=NA, p.me=NA, brt01=NA, brt02=NA, brt1=NA, brt2=NA,
p.brt=NA, Npres=NA)
n <- 0
for(s in 1:length(sp.data)){
  spi <- sp.data[[s]]

  # Select and calculate land cover predictors
  spi$forest <- spi$primf + spi$secdf
  spi$non_forest <-  spi$primn + spi$secdn
  spi$pasture <-  spi$pastr + spi$range
  spi$crops <- spi$c3ann + spi$c4ann + spi$c3per + spi$c4per + spi$c3nfx

  # Final set of variables
  spi <- spi[,c("pres","origin","bio_5","bio_6","bio_13","bio_14","bio_15",
              "forest","non_forest","pasture","crops","urban")]

  # Prepare data
  spi$pres[is.na(spi$pres)] <- 0
  nat <- spi[spi$origin=="native",]
  inv <- spi[spi$origin=="alien",]
  predictabil1[s,"Npres"] <- nrow(nat[nat$pres==1,])

  # Sample background points
  idp <- 1:nrow(nat)
  if(length(nat$pres[nat$pres==0])>10000){
    nat <- rbind(nat[nat$pres==1,], nat[sample(idp[nat$pres==0],10000, replace=F),])
  }

  # Standardise predictors
  for(v in 3:12){
    # Scale the training data and save the parameters
    nat[,v] <- scale(nat[,v])
    # Extract the scaling parameters
    train_center <- attr(nat[,v], "scaled:center")
    train_scale <- attr(nat[,v], "scaled:scale")
    # Apply the training parameters to the test data
    inv[,v] <- scale(inv[,v], center = train_center, scale = train_scale)
  }

  # Maxent
  #---------------
  occ <- nat$pres
  predictors <- nat[,3:12]
  me1 <- maxent(predictors, occ,
```

```
            args=c('linear=true', 'product=false', 'quadratic=true',
                 'hinge=false','threshold=false'))
me2 <- maxent(predictors, occ,
            args=c('linear=true', 'product=true', 'quadratic=true',
                 'hinge=false','threshold=false'))

# GOF
preds.me01 <- predict(me1, predictors)
preds.me02 <- predict(me2, predictors)
perf0.me01 <-  performance(prediction(preds.me01, occ),"auc")
perf.me01 <- as.numeric(perf0.me01@y.values)
perf0.me02 <-  performance(prediction(preds.me02, occ),"auc")
perf.me02 <- as.numeric(perf0.me02@y.values)

# Predictive ability of invasion
preds.me1 <- predict(me1, inv[,3:12])
preds.me2 <- predict(me2, inv[,3:12])
perf0.me1 <-  roc(inv$pres,preds.me1,quiet=T)
perf.me1 <- as.numeric(auc(perf0.me1))
perf0.me2 <-  roc(inv$pres,preds.me2,quiet=T)
perf.me2 <- as.numeric(auc(perf0.me2))
p.me <- roc.test(perf0.me1, perf0.me2)$p.value


# BRT
#---------------

set.seed(77)
brt1 <- gbm(nat$pres ~ ., data=nat[,3:12],
          distribution = "bernoulli",
          shrinkage = 0.01, # a.k.a. learning rate, or weight of each tree
          interaction.depth = 1,
          n.trees = 1000,
          bag.fraction = 0.75,
          cv.folds = 5,
          keep.data = TRUE,
          n.cores = 5)

set.seed(77)
brt2 <- gbm(nat$pres ~ ., data=nat[,3:12],
          distribution = "bernoulli",
          shrinkage = 0.01, # a.k.a. learning rate, or weight of each tree
          interaction.depth = 2,
          n.trees = 1000,
          bag.fraction = 0.75,
          cv.folds = 5,
          keep.data = TRUE,
```

```
            n.cores = 5)
  best.iter1 <- gbm.perf(brt1, method = "cv", plot.it = FALSE)
  best.iter2 <- gbm.perf(brt2, method = "cv", plot.it = FALSE)

  # GOF
  preds.brt01 <- predict(brt1, newdata = nat[,3:12], n.trees = best.iter1, type="response")
  preds.brt02 <- predict(brt2, newdata = nat[,3:12], n.trees = best.iter2, type="response")
  perf0.brt01 <-  performance(prediction(preds.brt01, nat$pres),"auc")
  perf.brt01 <- as.numeric(perf0.brt01@y.values)
  perf0.brt02 <-  performance(prediction(preds.brt02, nat$pres),"auc")
  perf.brt02 <- as.numeric(perf0.brt02@y.values)

  # Predictive ability of invasion
  preds.brt1 <- predict(brt1, newdata = inv[,3:12], n.trees = best.iter1, type="response")
  preds.brt2 <- predict(brt2, newdata = inv[,3:12], n.trees = best.iter2, type="response")
  perf0.brt1 <-  roc(inv$pres,preds.brt1,quiet=T)
  perf.brt1 <- as.numeric(auc(perf0.brt1))
  perf0.brt2 <-  roc(inv$pres,preds.brt2,quiet=T)
  perf.brt2 <- as.numeric(auc(perf0.brt2))
  p.brt <- roc.test(perf0.brt1, perf0.brt2)$p.value

  predictabil1[s,3:12] <- c(perf.me01, perf.me02, perf.me1, perf.me2, p.me,
                        perf.brt01, perf.brt02, perf.brt1, perf.brt2, p.brt)

  n <- n + 1
  print(n)
}

# Add geographical area of native occurrences (minimum convex polygon)
a <- c()
centr <- list()
for(s in 1:length(sp.data)){
  spi <- sp.data[[s]]
  nat <- spi[spi$origin=="native" & !is.na(spi$pres),]
  occ.sf <- st_as_sf(x = nat, coords = c("x", "y"), crs = "+proj=longlat +datum=WGS84")
  occ1 <- st_union(occ.sf)
  pol <- st_convex_hull(occ1)
  a[s] <- as.numeric(st_area(pol))/1e6
  centr[[s]] <- st_centroid(pol)
}

predictabil1$area <- a
centr1 <- do.call(rbind, lapply(centr, st_sf))

# Calculate proportion of non-overlap environmental conditions in origin vs invaded
env.new1 <- c()
for(s in 1:length(sp.data)){
```

```
  spi <- sp.data[[s]]

  # Select and calculate land cover predictors
  spi$forest <- spi$primf + spi$secdf
  spi$non_forest <-  spi$primn + spi$secdn
  spi$pasture <-  spi$pastr + spi$range
  spi$crops <- spi$c3ann + spi$c4ann + spi$c3per + spi$c4per + spi$c3nfx

  # Final set of variables
  spi <- spi[,c("pres","origin","bio_5","bio_6","bio_13","bio_14","bio_15",
            "forest","non_forest","pasture","crops","urban")]

  # Standardise predictors
  for(v in 3:12) spi[,v] <- scale(spi[,v])

  # Calculate reference parameters
  ref_center <- colMeans(spi[spi$origin=="native",3:12], na.rm=T)
  ref_covariance <- cov(spi[spi$origin=="native",3:12])
  df_count <- ncol(spi[spi$origin=="native",3:12])

  # Calculate Mahalanobis distance for all target points
  target_distances <- mahalanobis(x = spi[spi$origin=="alien",3:12], center = ref_center, cov =
ref_covariance)
  # Set your critical threshold (empirical quantile)
  critical_value <- quantile(target_distances, probs=0.95)
  # Calculate the proportion of target points exceeding this threshold
  env.new1[s] <- sum(target_distances > critical_value) / length(target_distances)
}


##########################################################################
##########################################################################

# With data from Elith et al 2020
# https://doi.org/10.17161/bi.v15i2.13384

# Code according to (using package "disdat")
# https://cran.r-project.org/web/packages/disdat/vignettes/modeling.html

library(disdat)

# provide names for regions to be modeled - here we model all 6:
regions <- c("AWT", "CAN", "NSW", "NZ", "SA", "SWI")
# specify names of all categorical variables across all regions:
categoricalvars <- c("ontveg", "vegsys", "toxicats", "age", "calc")
```

```
predictabil2 <- data.frame(region=NA, sp=NA,  me01=NA, me02=NA,me1=NA, me2=NA,
p.me=NA,
                      brt01=NA, brt02=NA, brt1=NA, brt2=NA, p.brt=NA, Npres=NA)

# Extract regions and species ID
n <- 0
for (r in regions) {
  # reading species data for this region, one
  # file per region:
  presences <- disPo(r)
  # extract names for all species
  species <- unique(presences$spid)
  for (s in species) {
    n <- n+1
    predictabil2[n,c(1,2)] <- c(r, s)
  }
}

# Fit models
for (z in 1:nrow(predictabil2)) {
  # reading presence-only and background species data for this region, one
  # file per region:
  r <- predictabil2$region[z]
  s <- predictabil2$sp[z]
  presences <- disPo(r)
  background <- disBg(r)

  # now for each species, prepare data for modeling and evaluation with
  # randomForests, model, predict to the evaluation file with environmental
  # data.
  # subset presence records of species for this species
  sp_presence <- presences[presences$spid == s, ]
  # add background data
  pr_bg <- rbind(sp_presence, background)

  # find the evaluation file – for some regions this means identifying
  # the taxonomic group
  if (r %in% c("AWT", "NSW")) {
    grp <- sp_presence[, "group"][1]
    evaluation <- disEnv(r, grp)
  } else {
    evaluation <- disEnv(r)
  }

  # convert categorical vars to factor in both training and testing data.
  # We use the package forcats to ensure that the levels of the factor in
  # the evaluation data match those in the training data, regardless of
```

```
# whether all levels are present in the evaluation data.
for (i in 1:ncol(pr_bg)) {
  if (colnames(pr_bg)[i] %in% categoricalvars) {
    fac_col <- colnames(pr_bg)[i]
    pr_bg[, fac_col] <- as.factor(pr_bg[, fac_col])
    evaluation[, fac_col] <- as.factor(evaluation[, fac_col])
    evaluation[, fac_col] <- forcats::fct_expand(evaluation[, fac_col],
                                                 levels(pr_bg[, fac_col]))
    evaluation[, fac_col] <- forcats::fct_relevel(evaluation[, fac_col],
                                                  levels(pr_bg[, fac_col]))
  }
}

# extract the relevant columns for modelling
model_data <- pr_bg[, c("occ", disPredictors(r))]
occ <- pr_bg[, "occ"]
predictors <- pr_bg[, disPredictors(r)]

# start modeling! We use the 'try' notation so if a species fails to
# fit, the loop will continue.

# Maxent
#---------------
if (inherits(try(me1 <- maxent(predictors, occ,
                              args=c('linear=true', 'product=false', 'quadratic=true',
                                     'hinge=false','threshold=false'))),
             "try-error")) {
  print(paste("Error for species", s, "from", r))
  next
}

if (inherits(try(me2 <- maxent(predictors, occ,
                              args=c('linear=true', 'product=true', 'quadratic=true',
                                     'hinge=false','threshold=false'))),
             "try-error")) {
  print(paste("Error for species", s, "from", r))
  next
}

# BRT
#---------------

set.seed(77)
if (inherits(try(brt1 <- gbm(model_data$occ ~ ., data=model_data[,disPredictors(r)],
                            distribution = "bernoulli",
                            shrinkage = 0.01, # a.k.a. learning rate, or weight of each tree
                            interaction.depth = 1,
```

```
                            n.trees = 1000,
                            bag.fraction = 0.75,
                            cv.folds = 5,
                            keep.data = TRUE,
                            n.cores = 5)),
            "try-error")) {
  print(paste("Error for species", s, "from", r))
  next
}

set.seed(77)
if (inherits(try(brt2 <- gbm(model_data$occ ~ ., data=model_data[,disPredictors(r)],
                            distribution = "bernoulli",
                            shrinkage = 0.01, # a.k.a. learning rate, or weight of each tree
                            interaction.depth = 2,
                            n.trees = 1000,
                            bag.fraction = 0.75,
                            cv.folds = 5,
                            keep.data = TRUE,
                            n.cores = 5)),
            "try-error")) {
  print(paste("Error for species", s, "from", r))
  next
}

# Performance

# Evaluation PA data
if (r %in% c("AWT", "NSW")) {
  grp <- sp_presence[, "group"][1]
  evals <- disPa(r, grp)
} else {
  evals <- disPa(r)
}

preds.me1 <- predict(me1, evaluation)
preds.me2 <- predict(me2, evaluation)
best.iter1 <- gbm.perf(brt1, method = "cv", plot.it = FALSE)
preds.brt1 <- predict(brt1, newdata = evaluation, n.trees = best.iter1, type="response")
best.iter2 <- gbm.perf(brt2, method = "cv", plot.it = FALSE)
preds.brt2 <- predict(brt2, newdata = evaluation, n.trees = best.iter2, type="response")

perf0.me1 <-  roc(evals[, s],preds.me1,quiet=T)
perf.me1 <- as.numeric(auc(perf0.me1))
perf0.me2 <-  roc(evals[, s],preds.me2,quiet=T)
perf.me2 <- as.numeric(auc(perf0.me2))
p.me <- roc.test(perf0.me1, perf0.me2)$p.value
```

```
  perf0.brt1 <-  roc(evals[, s],preds.brt1,quiet=T)
  perf.brt1 <- as.numeric(auc(perf0.brt1))
  perf0.brt2 <-  roc(evals[, s],preds.brt2,quiet=T)
  perf.brt2 <- as.numeric(auc(perf0.brt2))
  p.brt <- roc.test(perf0.brt1, perf0.brt2)$p.value

  # GOF
  preds.me01 <- predict(me1, predictors)
  preds.me02 <- predict(me2, predictors)
  perf0.me01 <-  performance(prediction(preds.me01, occ),"auc")
  perf.me01 <- as.numeric(perf0.me01@y.values)
  perf0.me02 <-  performance(prediction(preds.me02, occ),"auc")
  perf.me02 <- as.numeric(perf0.me02@y.values)

  preds.brt01 <- predict(brt1, newdata = model_data[,disPredictors(r)], n.trees = best.iter1,
type="response")
  preds.brt02 <- predict(brt2, newdata = model_data[,disPredictors(r)], n.trees = best.iter2,
type="response")
  perf0.brt01 <-  performance(prediction(preds.brt01, model_data$occ),"auc")
  perf.brt01 <- as.numeric(perf0.brt01@y.values)
  perf0.brt02 <-  performance(prediction(preds.brt02, model_data$occ),"auc")
  perf.brt02 <- as.numeric(perf0.brt02@y.values)

  Npres <- length(model_data$occ[model_data$occ==1])
  predictabil2[z,3:ncol(predictabil2)] <- c(perf.me01, perf.me02, perf.me1, perf.me2, p.me,
                     perf.brt01, perf.brt02, perf.brt1, perf.brt2, p.brt, Npres)
  print(z)
}


setwd("/Users/viana/Dropbox/Paper_niche_spatial-change")
save(predictabil2, file="predictabil2.2.RData")


# Add geographical area of native occurrences (minimum convex polygon)
a <- c()
rr <- c()
ss <- c()
centr <- list()
n <- 0
for (r in regions) {
  # reading presence-only and background species data for this region, one
  # file per region:
  presences <- disPo(r)

  # extract names for all species
  species <- unique(presences$spid)
```

```
  if (r=="AWT") {
    proj <- "+proj=utm +zone=55 +south +ellps=GRS80"
  } else if (r=="CAN") {
    proj <- "+proj=longlat +ellps=clrk66"
  } else if (r=="NZ") {
    proj <- "+proj=nzmg +lat_0=-41 +lon_0=173 +x_0=2510000 +y_0=6023150 +ellps=intl
+towgs84=59.47,-5.04,187.44,0.47,-0.1,1.024,-4.5993 +units=m"
  } else if (r=="SWI") {
    proj <- "+proj=tmerc +lat_0=46.95228333333333 +lon_0=7.439583333333333 +k=1
+x_0=600000 +y_0=200000 +ellps=bessel +units=m +no_defs"
  } else {
    proj <- "+proj=longlat +datum=WGS84"
  }

  # now for each species, prepare data for modeling and evaluation with
  # randomForests, model, predict to the evaluation file with environmental
  # data.
  for (s in species) {
    # subset presence records of species for this species
    nat <- presences[presences$spid == s, ]


    # start modeling! We use the 'try' notation so if a species fails to
    # fit, the loop will continue.
    n <- n + 1

    occ.sf <- st_as_sf(x = nat, coords = c("x", "y"), crs = proj)
    occ1 <- st_union(occ.sf)
    pol <- st_convex_hull(occ1)
    centrn <- st_centroid(pol)
    centr[[n]] <- st_transform(centrn, crs="+proj=longlat +datum=WGS84")
    a[n] <- as.numeric(st_area(pol))/1e6
    rr[n] <- r
    ss[n] <- s
  }
}

predictabil2$id <- paste(predictabil2$region, predictabil2$sp, sep="_")
a.df <- data.frame(id=paste(rr, ss, sep="_"), area=a)
predictabil2 <- left_join(predictabil2, a.df, by="id")
predictabil2 <- predictabil2[,-14]

centr2 <- do.call(rbind, lapply(centr, st_sf))


# Calculate proportion of non-overlap environmental conditions in origin vs invaded
```

```
env.new2 <- c()
n <- 0
for (r in regions) {
  # reading presence-only and background species data for this region, one
  # file per region:
  presences <- disPo(r)
  background <- disBg(r)

  # extract names for all species
  species <- unique(presences$spid)

  # now for each species, prepare data for modeling and evaluation with
  # randomForests, model, predict to the evaluation file with environmental
  # data.
  for (s in species) {
    # subset presence records of species for this species
    sp_presence <- presences[presences$spid == s, ]
    # add background data
    pr_bg <- rbind(sp_presence, background)

    # find the evaluation file – for some regions this means identifying
    # the taxonomic group
    if (r %in% c("AWT", "NSW")) {
      grp <- sp_presence[, "group"][1]
      evaluation <- disEnv(r, grp)
    } else {
      evaluation <- disEnv(r)
    }

    # convert categorical vars to factor in both training and testing data.
    # We use the package forcats to ensure that the levels of the factor in
    # the evaluation data match those in the training data, regardless of
    # whether all levels are present in the evaluation data.
    for (i in 1:ncol(pr_bg)) {
      if (colnames(pr_bg)[i] %in% categoricalvars) {
        fac_col <- colnames(pr_bg)[i]
        pr_bg[, fac_col] <- as.factor(pr_bg[, fac_col])
        evaluation[, fac_col] <- as.factor(evaluation[, fac_col])
        evaluation[, fac_col] <- forcats::fct_expand(evaluation[, fac_col],
                                            levels(pr_bg[, fac_col]))
        evaluation[, fac_col] <- forcats::fct_relevel(evaluation[, fac_col],
                                             levels(pr_bg[, fac_col]))
      }
    }

    # extract the relevant columns for modelling
    predictors.train <- pr_bg[, disPredictors(r)]
```

```
    v.in <- sapply(predictors.train, class)
    predictors.train <- predictors.train[,v.in %in% c("numeric","integer")]
    predictors.eval <- evaluation[,5:ncol(evaluation)]
    predictors.eval <- predictors.eval[,v.in %in% c("numeric","integer")]

    # start modeling! We use the 'try' notation so if a species fails to
    # fit, the loop will continue.
    n <- n + 1

    # Calculate reference parameters
    try({ref_center <- colMeans(predictors.train, na.rm=T)
    ref_covariance <- cov(predictors.train)
    diag(ref_covariance) <- diag(ref_covariance) + 1e-6
    df_count <- ncol(predictors.train)

    # Calculate Mahalanobis distance for all target points
    target_distances <- mahalanobis(x = predictors.eval, center = ref_center, cov =
ref_covariance)
    # Set your critical threshold (e.g., 95% confidence boundary)
    critical_value <- quantile(target_distances, probs=0.95)
    # Calculate the proportion of target points exceeding this threshold
    env.new2[n] <- sum(target_distances > critical_value) / length(target_distances)
    })
  }
}

##########################################################################
##########################################################################

# With data from Atwater & Barney (plants)
#  https://doi.org/10.1111/geb.13342

a <- read.csv("025-PointData-AllValid-RPCA.csv")
asp <- unique(a$species)

predictabil3 <- data.frame(sp=asp, me01=NA, me02=NA, me1=NA, me2=NA, p.me=NA,
                          brt01=NA, brt02=NA, brt1=NA, brt2=NA, p.brt=NA, Npres=NA)

for(s in 1:length(asp)){
  spi <- a[a$species==asp[s],]
  if(length(spi$invasive[spi$invasive==0])<20) next
  if(length(spi$invasive[spi$invasive==1])<10) next
  bg.df <- a[a$species!=asp[s],]
  bg.df <- bg.df[!is.na(bg.df$region),]
  bg.df.nat <- bg.df[bg.df$region %in% na.exclude(unique(spi$region[spi$invasive==0])),]
  bg <- bg.df.nat[sample(1:nrow(bg.df.nat), 10000),]
  bg.df.inv <- bg.df[bg.df$region %in% na.exclude(unique(spi$region[spi$invasive==1])),]
```

```r
bg.i <- bg.df.inv[sample(1:nrow(bg.df.inv), length(spi$invasive[spi$invasive==1])),]

# Prepare data
nat <- rbind(spi[spi$invasive==0,],bg)
nat$pres <- c(rep(1,nrow(spi[spi$invasive==0,])),rep(0,nrow(bg)))
inv <- rbind(spi[spi$invasive==1,],bg.i)
inv$pres <- c(rep(1,nrow(spi[spi$invasive==1,])),rep(0,nrow(bg.i)))

# Maxent
#---------------
occ <- nat$pres
predictors <- nat[,c("RCA1","RCA2","RCA3","RCA4")]
me1 <- maxent(predictors, occ,
           args=c('linear=true', 'product=false', 'quadratic=true',
                'hinge=false','threshold=false'))
me2 <- maxent(predictors, occ,
           args=c('linear=true', 'product=true', 'quadratic=true',
                'hinge=false','threshold=false'))

# GOF
preds.me01 <- predict(me1, predictors)
preds.me02 <- predict(me2, predictors)
perf0.me01 <-  performance(prediction(preds.me01, occ),"auc")
perf.me01 <- as.numeric(perf0.me01@y.values)
perf0.me02 <-  performance(prediction(preds.me02, occ),"auc")
perf.me02 <- as.numeric(perf0.me02@y.values)

# Predictive ability of invasion
preds.me1 <- predict(me1, inv[,c("RCA1","RCA2","RCA3","RCA4")])
preds.me2 <- predict(me2, inv[,c("RCA1","RCA2","RCA3","RCA4")])
perf0.me1 <-  roc(inv$pres,preds.me1,quiet=T)
perf.me1 <- as.numeric(auc(perf0.me1))
perf0.me2 <-  roc(inv$pres,preds.me2,quiet=T)
perf.me2 <- as.numeric(auc(perf0.me2))
p.me <- roc.test(perf0.me1, perf0.me2)$p.value

# BRT
#---------------

set.seed(77)
brt1 <- gbm(nat$pres ~ ., data=nat[,c("RCA1","RCA2","RCA3","RCA4")],
          distribution = "bernoulli",
          shrinkage = 0.01, # a.k.a. learning rate, or weight of each tree
          interaction.depth = 1,
          n.trees = 1000,
          bag.fraction = 0.75,
          cv.folds = 5,
```

```
          keep.data = TRUE,
          n.cores = 5)

  set.seed(77)
  brt2 <- gbm(nat$pres ~ ., data=nat[,c("RCA1","RCA2","RCA3","RCA4")],
          distribution = "bernoulli",
          shrinkage = 0.01, # a.k.a. learning rate, or weight of each tree
          interaction.depth = 2,
          n.trees = 1000,
          bag.fraction = 0.75,
          cv.folds = 5,
          keep.data = TRUE,
          n.cores = 5)
  best.iter1 <- gbm.perf(brt1, method = "cv", plot.it = FALSE)
  best.iter2 <- gbm.perf(brt2, method = "cv", plot.it = FALSE)

  # GOF
  preds.brt01 <- predict(brt1, newdata = nat[,c("RCA1","RCA2","RCA3","RCA4")], n.trees =
best.iter1, type="response")
  preds.brt02 <- predict(brt2, newdata = nat[,c("RCA1","RCA2","RCA3","RCA4")], n.trees =
best.iter2, type="response")
  perf0.brt01 <-  performance(prediction(preds.brt01, nat$pres),"auc")
  perf.brt01 <- as.numeric(perf0.brt01@y.values)
  perf0.brt02 <-  performance(prediction(preds.brt02, nat$pres),"auc")
  perf.brt02 <- as.numeric(perf0.brt02@y.values)

  # Predictive ability of invasion
  preds.brt1 <- predict(brt1, newdata = inv[,c("RCA1","RCA2","RCA3","RCA4")], n.trees =
best.iter1, type="response")
  preds.brt2 <- predict(brt2, newdata = inv[,c("RCA1","RCA2","RCA3","RCA4")], n.trees =
best.iter2, type="response")
  perf0.brt1 <-  roc(inv$pres,preds.brt1,quiet=T)
  perf.brt1 <- as.numeric(auc(perf0.brt1))
  perf0.brt2 <-  roc(inv$pres,preds.brt2,quiet=T)
  perf.brt2 <- as.numeric(auc(perf0.brt2))
  p.brt <- roc.test(perf0.brt1, perf0.brt2)$p.value

  Npres <- length(spi$invasive[spi$invasive==0])
  predictabil3[s,] <- c(asp[s], perf.me01, perf.me02, perf.me1, perf.me2, p.me,
                    perf.brt01, perf.brt02, perf.brt1, perf.brt2, p.brt, Npres)
  print(s)
}

setwd("/Users/viana/Dropbox/Paper_niche_spatial-change")
save(predictabil3, file="predictabil3.2.RData")

# Add geographical area of native occurrences (minimum convex polygon)
```

```
aa <- c()
centr <- list()
for(s in 1:length(asp)){
  spi <- a[a$species==asp[s],]
  if(length(spi$invasive[spi$invasive==0])<20) next
  if(length(spi$invasive[spi$invasive==1])<10) next
  nat <- spi[spi$invasive==0,]
  occ.sf <- st_as_sf(x = nat, coords = c("lon", "lat"), crs = "+proj=longlat +datum=WGS84")
  occ1 <- st_union(occ.sf)
  pol <- st_convex_hull(occ1)
  centr[[s]] <- st_centroid(pol)
  aa[s] <- as.numeric(st_area(pol))/1e6
}

predictabil3$area <- aa

centr <- centr[which(!is.na(predictabil3$Npres))]
centr3 <- do.call(rbind, lapply(centr, st_sf))


# Calculate proportion of non-overlap environmental conditions in origin vs invaded
env.new3 <- c()
for(s in 1:length(asp)){
  spi <- a[a$species==asp[s],]
  if(length(spi$invasive[spi$invasive==0])<20) next
  if(length(spi$invasive[spi$invasive==1])<10) next
  bg.df <- a[a$species!=asp[s],]
  bg.df <- bg.df[!is.na(bg.df$region),]
  bg.df.nat <- bg.df[bg.df$region %in% na.exclude(unique(spi$region[spi$invasive==0])),]
  bg <- bg.df.nat[sample(1:nrow(bg.df.nat), 10000),]
  bg.df.inv <- bg.df[bg.df$region %in% na.exclude(unique(spi$region[spi$invasive==1])),]
  bg.i <- bg.df.inv[sample(1:nrow(bg.df.inv), length(spi$invasive[spi$invasive==1])),]

  # Prepare data
  nat <- rbind(spi[spi$invasive==0,],bg)
  inv <- rbind(spi[spi$invasive==1,],bg.i)

  # Calculate reference parameters
  try({ref_center <- colMeans(nat[,7:10], na.rm=T)
  ref_covariance <- cov(nat[,7:10])
  diag(ref_covariance) <- diag(ref_covariance) + 1e-6
  df_count <- ncol(nat[,7:10])

  # Calculate Mahalanobis distance for all target points
  target_distances <- mahalanobis(x = inv[,7:10], center = ref_center, cov = ref_covariance)
  # Set your critical threshold (e.g., 95% confidence boundary)
  critical_value <- quantile(target_distances, probs=0.95)
```

```
  # Calculate the proportion of target points exceeding this threshold
  env.new3[s] <- sum(target_distances > critical_value) / length(target_distances)
  })
}


########################################################################
########################################################################

# Model comparison

na1 <- which(is.na(predictabil1$me01))
na3 <- which(is.na(predictabil3$me01))
predictabil1 <- predictabil1[-na1,]
predictabil3 <- predictabil3[-na3,]
centr1 <- centr1[-na1,]
env.new1 <- env.new1[-na1]

for(i in 3:ncol(predictabil2)) predictabil2[,i] <- as.numeric(predictabil2[,i])
for(i in 2:ncol(predictabil3)) predictabil3[,i] <- as.numeric(predictabil3[,i])

p1.in <- which(predictabil1$me01>=0.70 | predictabil1$me02>=0.70 |
               predictabil1$brt01>=0.70 | predictabil1$brt02>=0.70)
predictabil1 <- predictabil1[p1.in,]
p2.in <- which(predictabil2$me01>=0.70 | predictabil2$me02>=0.70 |
               predictabil2$brt01>=0.70 | predictabil2$brt02>=0.70)
predictabil2 <- predictabil2[p2.in,]
p3.in <- which(predictabil3$me01>=0.70 | predictabil3$me02>=0.70 |
               predictabil3$brt01>=0.70 | predictabil3$brt02>=0.70)
predictabil3 <- predictabil3[p3.in,]

centr1 <- centr1[p1.in,]
centr2 <- centr2[p2.in,]
centr3 <- centr3[p3.in,]
env.new1 <- env.new1[p1.in]
env.new2 <- env.new2[p2.in]
env.new3 <- env.new3[p3.in]

p1 <- data.frame(type="invasion",predictabil1[,3:ncol(predictabil1)])
p1$taxon <- "birds"
p1$study <- "Inv"
p1$dataset <- "Exotic_birds"
p2 <- data.frame(type="independent",predictabil2[,3:ncol(predictabil2)])
p2$taxon <- c(rep("birds",20),rep("plants",20),rep("birds",20),rep("bats",7),

rep("birds",10),rep("plants",29),rep("reptiles",8),rep("plants",52),rep("plants",30),rep("plant
s",30))
```

```
p2$study <- "Ind"
p2$dataset <- "Native_area"
p3 <- data.frame(type="invasion",predictabil3[,2:ncol(predictabil3)])
p3$taxon <- "plants"
p3$study <- "Inv"
p3$dataset <- "Exotic_plants"
p0 <- rbind(p1,p2,p3)
#p0 <- p0[,-c(5,6)]
p0$me <- p0$me2-p0$me1
p0$brt <- p0$brt2-p0$brt1
pw <- p0[,c("type","me","brt","Npres","area","taxon","study","dataset")]
pw$lat <- abs(c(st_coordinates(centr1)[,2], st_coordinates(centr2)[,2],
st_coordinates(centr3)[,2]))
pw$env.new <- c(env.new1, env.new2, env.new3)
pl <- pivot_longer(pw,cols=me:brt, names_to = "model", values_to = "AUCdiff")
pl$Npres <- log(pl$Npres)
pl$area <- log(pl$area)
pl <- as.data.frame(pl)
pl$study_taxon <- paste(pl$study,pl$taxon,sep="_")
pl$dataset <- as.factor(pl$dataset)
pl$dataset <- relevel(pl$dataset, ref = "Native_area")

# Drivers of model transferability
#-----------------------------------------
mc <- lm(AUCdiff ~ dataset * model + Npres + env.new + area, data=pl)
summary(mc)
anova(mc)

# Model for native area
mc1 <- lm(AUCdiff ~ model*taxon + Npres + env.new + area,
data=pl[pl$dataset=="Native_area",])
summary(mc1)
anova(mc1)
# Model for exotic birds
mc2 <- lm(AUCdiff ~ model + Npres + env.new + area, data=pl[pl$dataset=="Exotic_birds",])
summary(mc2)
anova(mc2)
# Model for exotic plants
mc3 <- lm(AUCdiff ~ model + Npres + env.new + area, data=pl[pl$dataset=="Exotic_plants",])
summary(mc3)
anova(mc3)

# Only BRT
# Model for native area
mc1 <- lm(AUCdiff ~ taxon + Npres + env.new + area,
data=pl[pl$dataset=="Native_area"&pl$model=="brt",])
summary(mc1)
```

```
anova(mc1)
# Model for exotic birds
mc2 <- lm(AUCdiff ~ Npres + env.new + area,
data=pl[pl$dataset=="Exotic_birds"&pl$model=="brt",])
summary(mc2)
anova(mc2)
# Model for exotic plants
mc3 <- lm(AUCdiff ~ Npres + env.new + area,
data=pl[pl$dataset=="Exotic_plants"&pl$model=="brt",])
summary(mc3)
anova(mc3)

# Only MaxEnt
# Model for native area
mc1 <- lm(AUCdiff ~ taxon + Npres + env.new + area,
data=pl[pl$dataset=="Native_area"&pl$model=="me",])
summary(mc1)
anova(mc1)
# Model for exotic birds
mc2 <- lm(AUCdiff ~ Npres + env.new + area,
data=pl[pl$dataset=="Exotic_birds"&pl$model=="me",])
summary(mc2)
anova(mc2)
# Model for exotic plants
mc3 <- lm(AUCdiff ~ Npres + env.new + area,
data=pl[pl$dataset=="Exotic_plants"&pl$model=="me",])
summary(mc3)
anova(mc3)


# Mean predictive performance
apply(p0[,c(4,5,9,10)],2,median)
quantile(unlist(p0[,c(4,5,9,10)]), probs = c(0.05, 0.5, 0.95))
range(unlist(p0[,c(4,5,9,10)]))
median(unlist(p0[,4:5]))
median(unlist(p0[,9:10]))

# Correlation between performance of the two model types (MaxEnt and BRT)
cor.test(p0$me1,p0$brt1)
cor.test(p0$me2,p0$brt2)
cor.test(p0$me,p0$brt)

# Compare models with and without interactions
library(EnvStats)
boxplot(p0[,c(4,5,9,10)])
twoSamplePermutationTestLocation(c(p0$me1,p0$brt1), c(p0$me2,p0$brt2), paired =
TRUE)
```

```
twoSamplePermutationTestLocation(p0$me1, p0$brt1, paired = TRUE)
twoSamplePermutationTestLocation(p0$me2, p0$brt2, paired = TRUE)

# Mean AUC difference
mean(p0$me); sd(p0$me)
mean(p0$brt); sd(p0$brt)

# Significant improvement or worsening
nrow(p0[p0$me>0 & p0$p.me<0.05,]) / nrow(p0) # % improvement w/ ME
nrow(p0[p0$me<0 & p0$p.me<0.05,]) / nrow(p0) # % worsening w/ ME
nrow(p0[p0$brt>0 & p0$p.brt<0.05,]) / nrow(p0) # % improvement w/ BRT
nrow(p0[p0$brt<0 & p0$p.brt<0.05,]) / nrow(p0) # % worsening w/ BRT
nrow(p0[p0$me>0 & p0$brt>0 & p0$p.me<0.05 & p0$p.brt<0.05,]) / nrow(p0) # %
consistent improvement
nrow(p0[p0$me<0 & p0$brt<0 & p0$p.me<0.05 & p0$p.brt<0.05,]) / nrow(p0) # %
consistent worsening

#########################################################################
#########################################################################

# Figures

library(ggplot2)
theme_set(theme_classic())
library(scales)
library(cowplot)
library(egg)
library(maps)
library(mapdata)

# Figure 1: map of training centroids

quartz(height=4, width=7)
par(mar=c(0,0,0,0))
map('worldHires')
plot(centr3, add=T, pch=16, col=alpha("green3", 0.5))
plot(centr1, add=T, pch=16, col=alpha("blue", 0.5))
plot(centr2, add=T, pch=16, col=alpha("violetred", 0.5))

quartz(height=2, width=3)
ggplot(data = data.frame(env.new1), aes(x = env.new1)) +
  geom_histogram(fill = "blue", color="black", binwidth = 0.005, center=0.0025) +
  coord_cartesian(xlim = c(0, 0.1)) +
  xlab("Prop. novel environment") + ylab("Frequency")

quartz(height=2, width=3)
ggplot(data = data.frame(env.new2), aes(x = env.new2)) +
```

```
  geom_histogram(fill = "violetred", color="black", binwidth = 0.005, center=0.0025) +
  coord_cartesian(xlim = c(0, 0.1)) +
  xlab("Prop. novel environment") + ylab("Frequency")

quartz(height=2, width=3)
ggplot(data = data.frame(env.new3), aes(x = env.new3)) +
  geom_histogram(fill = "green3", color="black", binwidth = 0.005, center=0.0025) +
  coord_cartesian(xlim = c(0, 0.1)) +
  xlab("Prop. novel environment") + ylab("Frequency")

#--------------------

# Figure 2: Maxent vs BRT

gg2.1 <- ggplot(p0, aes(x=me1, y=brt1)) +
  geom_point(size=1, shape=1) +
  geom_abline (slope=1, linetype = "dashed") +
  xlab("AUC MaxEnt") + ylab("AUC BRT")

gg2.2 <- ggplot(p0, aes(x=me2, y=brt2)) +
  geom_point(size=1, shape=1) +
  geom_abline (slope=1, linetype = "dashed") +
  xlab("AUC MaxEnt") + ylab("AUC BRT")

gg2.3 <- ggplot(p0, aes(x=me, y=brt)) +
  geom_point(size=1, shape=1) +
  geom_abline (slope=1, linetype = "dashed") +
  geom_hline (yintercept=0, linetype = "dashed") +
  geom_vline (xintercept=0, linetype = "dashed") +
  xlab("AUC MaxEnt") + ylab("AUC BRT")


quartz(height=3.5,width=10)
ggarrange(gg2.1, gg2.2, gg2.3, nrow=1, labels = c('(a)', '(b)', '(c)'))

#--------------------

# Figure 3: interaction predictability comparison
pl2 <- pivot_longer(p0,cols=c(brt1,brt2,me1,me2), names_to = "model", values_to = "AUC")
gg3.1 <- ggplot(pl2) +
  geom_boxplot(aes(model, AUC), width=0.5) +
  scale_x_discrete(labels= c("BRT_no-int","BRT_int","MaxEnt_no-int","MaxEnt_int")) +
  xlab("Model") + ylab("AUC")

pl$study_taxon <- paste(pl$study,pl$taxon,sep="_")
pl$model <- factor(pl$model)
levels(pl$model) <- c("BRT","MaxEnt")
```

```
gg3.2 <- ggplot(pl,aes(study_taxon, AUCdiff)) +
  geom_boxplot(aes(fill=model)) +
  geom_hline (yintercept=0, linetype = "dashed") +
  xlab("Data type and taxon") + ylab("AUC difference") +
  theme(legend.position = c(0.2, 0.8))

quartz(height=3.5,width=9)
ggarrange(gg3.1, gg3.2, nrow=1, labels = c('(a)', '(b)'))
```
