# Supplementary material for: Interaction Effects Do Not Consistently Improve the Transferability of Species Distribution Models
Source: Ecol Evol. 2026 Sep 13;16(9):e74362. doi: 10.1002/ece3.74362 (PMC13572786; doi:10.1002/ece3.74362)
Supplement: Supplementary file 2 [file ECE3-16-e74362-s002.docx]

**Supporting Information**

Supporting tables from:

*Interaction effects do not consistently improve the transferability of species distribution models*

Duarte S. Viana, Laura Cardador, Miguel Clavero

**Table S1**. Results of the model of AUC difference for the first case study (testing with independent data from native range) (R^2^ = 0.053). BRT models and the bats taxon are the reference levels (intercept) for the model type and taxon effects, respectively. The colon indicates an interaction effect.

| **Model term** | **Estimate ± SE** | **t-value** | **p-value** |
| --- | --- | --- | --- |
| Intercept | -0.118 ± 0.032 | -3.732 | 0.000* |
| Model_MaxEnt | -0.013 ± 0.015 | -0.824 | 0.410 |
| Taxon_birds | -0.019 ± 0.012 | -1.624 | 0.105 |
| Taxon_plants | -0.011 ± 0.011 | -1.016 | 0.310 |
| Taxon_reptiles | 0.011 ± 0.015 | 0.775 | 0.439 |
| Number_presences | 0.003 ± 0.001 | 3.134 | 0.002* |
| Prop_novel_environment | 2.362 ± 0.560 | 4.217 | 0.000* |
| Area | 0.000 ± 0.000 | 0.004 | 0.997 |
| Model_MaxEnt : Taxon_birds | 0.013 ± 0.016 | 0.779 | 0.437 |
| Model_MaxEnt : Taxon_plants | 0.012 ± 0.016 | 0.751 | 0.453 |
| Model_MaxEnt : Taxon_reptiles | -0.013 ± 0.021 | -0.646 | 0.519 |

**Table S2**. Results of the model of AUC difference for the second case study (testing with data from plants’ exotic range) (R^2^ = 0.003). BRT models is the reference level (intercept) for the model type effect.

| **Model term** | **Estimate ± SE** | **t-value** | **p-value** |
| --- | --- | --- | --- |
| Intercept | -0.054 ± 0.024 | -2.256 | 0.024* |
| Model_MaxEnt | -0.004 ± 0.003 | -1.357 | 0.175 |
| Number_presences | -0.001 ± 0.001 | -0.814 | 0.416 |
| Prop_novel_environment | 0.677 ± 0.319 | 2.125 | 0.034* |
| Area | 0.001 ± 0.001 | 1.225 | 0.221 |

**Table S3**. Results of the model of AUC difference for the third case study (testing with data from birds’ exotic range) (R^2^ = 0.000). BRT models is the reference level (intercept) for the model type effect.

| **Model term** | **Estimate ± SE** | **t-value** | **p-value** |
| --- | --- | --- | --- |
| Intercept | 0.039 ± 0.059 | 0.657 | 0.512 |
| Model_MaxEnt | 0.003 ± 0.009 | 0.347 | 0.729 |
| Number_presences | 0.005 ± 0.003 | 1.489 | 0.137 |
| Prop_novel_environment | -0.084 ± 0.477 | -0.177 | 0.860 |
| Area | -0.005 ± 0.004 | -1.108 | 0.269 |

**Table S4**. Results of the model of AUC difference resulting from BRT predictions for the first case study (testing with independent data from native range) (R^2^ = 0.107). The bats taxon is the reference level (intercept) for the taxon effects.

| **Model term** | **Estimate ± SE** | **t-value** | **p-value** |
| --- | --- | --- | --- |
| Intercept | -0.243 ± 0.054 | -4.487 | 0.000 |
| Taxon_birds | -0.018 ± 0.015 | -1.246 | 0.214 |
| Taxon_plants | -0.013 ± 0.014 | -0.954 | 0.341 |
| Taxon_reptiles | 0.011 ± 0.018 | 0.592 | 0.554 |
| Number_presences | 0.004 ± 0.002 | 2.317 | 0.021* |
| Prop_novel_environment | 4.896 ± 0.986 | 4.966 | 0.000* |
| Area | -0.001 ± 0.001 | -0.879 | 0.380 |

**Table S5**. Results of the model of AUC difference resulting from BRT predictions for the second case study (testing with data from plants’ exotic range) (R^2^ = 0.015).

| **Model term** | **Estimate ± SE** | **t-value** | **p-value** |
| --- | --- | --- | --- |
| Intercept | -0.115 ± 0.038 | -3.017 | 0.003* |
| Number_presences | 0.002 ± 0.001 | 1.218 | 0.224 |
| Prop_novel_environment | 1.368 ± 0.510 | 2.681 | 0.008* |
| Area | 0.002 ± 0.002 | 1.054 | 0.292 |

**Table S6**. Results of the model of AUC difference resulting from BRT predictions for the third case study (testing with data from birds’ exotic range) (R^2^ = 0.050).

| **Model term** | **Estimate ± SE** | **t-value** | **p-value** |
| --- | --- | --- | --- |
| Intercept | 0.061 ± 0.070 | 0.874 | 0.384 |
| Number_presences | 0.010 ± 0.004 | 2.501 | 0.014* |
| Prop_novel_environment | -1.111 ± 0.570 | -1.951 | 0.053 |
| Area | -0.005 ± 0.005 | -1.094 | 0.276 |

**Table S7**. Results of the model of AUC difference resulting from MaxEnt predictions for the first case study (testing with independent data from native range) (R^2^ = 0.063). The bats taxon is the reference level (intercept) for the taxon effects.

| **Model term** | **Estimate ± SE** | **t-value** | **p-value** |
| --- | --- | --- | --- |
| Intercept | -0.006 ± 0.026 | -0.236 | 0.814 |
| Taxon_birds | -0.007 ± 0.007 | -0.975 | 0.331 |
| Taxon_plants | 0.002 ± 0.007 | 0.364 | 0.716 |
| Taxon_reptiles | -0.001 ± 0.009 | -0.166 | 0.868 |
| Number_presences | 0.002 ± 0.001 | 2.591 | 0.010* |
| Prop_novel_environment | -0.171 ± 0.473 | -0.361 | 0.719 |
| Area | 0.001 ± 0.000 | 1.840 | 0.067 |

**Table S8**. Results of the model of AUC difference resulting from MaxEnt predictions for the second case study (testing with data from plants’ exotic range) (R^2^ = 0.012).

| **Model term** | **Estimate ± SE** | **t-value** | **p-value** |
| --- | --- | --- | --- |
| Intercept | 0.003 ± 0.028 | 0.102 | 0.919 |
| Number_presences | -0.003 ± 0.001 | -3.034 | 0.003* |
| Prop_novel_environment | -0.014 ± 0.376 | -0.037 | 0.970 |
| Area | 0.001 ± 0.001 | 0.646 | 0.519 |

**Table S9**. Results of the model of AUC difference resulting from MaxEnt predictions for the third case study (testing with data from birds’ exotic range) (R^2^ = 0.000).

| **Model term** | **Estimate ± SE** | **t-value** | **p-value** |
| --- | --- | --- | --- |
| Intercept | 0.019 ± 0.093 | 0.206 | 0.837 |
| Number_presences | 0.000 ± 0.005 | -0.007 | 0.995 |
| Prop_novel_environment | 0.943 ± 0.753 | 1.252 | 0.213 |
| Area | -0.004 ± 0.007 | -0.575 | 0.566 |
